# Effectiveness of family-centered communication on caregiving ability and health outcomes in premature infants: a systematic review protocol

**DOI:** 10.1186/s13643-026-03240-8

**Published:** 2026-06-15

**Authors:** Mira Miraturrofi’ah, Qorinah Estiningtyas Sakilah Adnani, Dwi Agustian, Dany Hilmanto

**Affiliations:** 1 Doctoral Study Program in Medical Science, Faculty of Medicine, Universitas Padjadjaran, Bandung, Indonesia; 2 Department of Midwifery, Faculty of Health Sciences, Universitas Bhakti Kencana, Bandung, Indonesia; 3 Department of Public Health, Faculty of Medicine, Universitas Padjadjaran, Bandung, Indonesia; 4 Department of Child Health, Faculty of Medicine, Universitas Padjadjaran, Bandung, Indonesia

**Keywords:** Caregiving ability, Family-centered communication, Infant health outcomes, Healthcare and community setting, Preterm infants, A systematic review protocol

## Abstract

**Background:**

Preterm neonatal morbidity and mortality remain major global health challenges, particularly in low-resource settings. Family-centered communication (FCC) has shown promise in strengthening caregiver competencies and improving neonatal outcomes. However, evidence on the effectiveness of FCC across diverse healthcare and community contexts, intervention modalities, and outcome measures remains fragmented.

**Methods:**

This systematic review protocol will adhere to the Preferred Reporting Items for Systematic Reviews and Meta-Analyses Protocols (PRISMA-P) guidelines. The review will include studies evaluating FCC interventions directed at parents or primary caregivers of preterm infants, excluding those with medical comorbidities. Eligible interventions will be delivered by healthcare professionals and include structured health education, counseling, communication training, multidisciplinary collaboration, and caregiver involvement in decision-making. Eligible study designs include randomized controlled trials, quasi-experimental, and analytical observational studies. The search strategy will be guided by the Population, Intervention, Comparator, Outcome (PICO) framework and conducted in PubMed, EMBASE, CINAHL, PsycINFO and Scopus, supplemented by manual screening of reference lists and key journals. Two reviewers will independently conduct study selection, critical appraisal, and data extraction. The findings will be synthesized narratively following the Synthesis Without Meta-analysis (SWiM) guidelines, and where appropriate, meta-analysis will be performed. Certainty of evidence will be appraised using the GRADE (Grading of Recommendations, Assessment, Development and Evaluation) approach, and results summarized in a Summary of Findings table.

**Discussion:**

This review aims to provide a comprehensive synthesis of evidence on the effectiveness of FCC strategies compared with standard care in improving caregivers’ ability and the health outcomes of preterm infants. The findings are expected to inform evidence-based practices and guide healthcare professionals, policymakers, and program developers in implementing effective FCC approaches across diverse settings.

**Systematic review registration:**

PROSPERO CRD420250645221

**Supplementary Information:**

The online version contains supplementary material available at 10.1186/s13643-026-03240-8.

## Background

Preterm birth is defined as birth occurring before 37 completed weeks of gestation and is commonly classified as extremely preterm (<28 weeks), very preterm (28 to <32 weeks), and moderate to late preterm (32 to <37 weeks), in line with WHO definitions [1, 2], and it continues to be a significant public health issue. This is due to the anatomical and physiological immaturity of these infants [3–5], which often manifests as low birth weight and an increased risk of long-term health complications [6]. Global trends suggest a sustained increase in preterm birth rates across all regions, from international to local levels [1]. According to data from the World Health Organization (WHO) and the United Nations Children’s Fund (UNICEF), the global preterm birth rate in 2020 ranged from 4 to 16% [1]. In 2019, an estimated 900,000 children under 4 years of age died as a result of preterm birth, and in 2020, approximately 1 million preterm infants died, while many who survived experienced lifelong disabilities [1]. Preterm birth increases the risk of chronic issues such as poor growth, cognitive delay, and motor deficits into adulthood [6]. These ongoing health challenges are frequently associated with increased hospital readmissions, thereby placing additional financial and logistical demands on families and healthcare systems. Evidence from different regions demonstrates substantial variation in preterm infant readmission rates across health system contexts. In Pakistan, a study at Sheikh Saeed Memorial Hospital in Karachi reported hospital readmission in 49% of 511 preterm infants, primarily due to infection and jaundice, with a mortality rate of 5.5% [7]. In a tertiary referral hospital in Northern India, the readmission rate was 16.4% [8]. In contrast, studies from California reported 30-day readmission rates ranging from 5.9% (7243 of 122,014 preterm infants) [9, 10], to approximately 8% [11], while a population-based study in Croatia documented a readmission rate of 4%, mainly related to respiratory infections, jaundice, and urinary tract infections [12].

These marked disparities highlight the influence of post-discharge care continuity, caregiver preparedness, and health system capacity on readmission risk, particularly in low- and middle-income countries.

Preterm infants need more care than full-term newborns do. While interventions for preterm infants in hospital settings often lead to positive results owing to the direct delivery of specialized care by healthcare professionals within the facility, challenges frequently emerge concerning discharge education and the establishment of effective postdischarge care routines at home [13]. Deficiencies in caregiver knowledge, reduced self-efficacy, and limited practical skills have been associated with suboptimal home care outcomes and increased likelihood of rehospitalization among preterm infants [10, 12, 14]. Research in Karachi's Neonatal Intensive Care Unit (NICU) revealed that 55 of 511 discharged newborns were readmitted, mostly for prematurity [7]. Overall, 25–50% of medically fragile infants are readmitted within the first year of life; 7.4% of preterm infants are readmitted within 2 weeks after discharge, and a separate retrospective cohort study conducted in a neonatal intensive care unit reported a readmission rate of 16.4% [8]. These largely preventable returns—often owing to feeding and care gaps—threaten infant growth and development and underscore the need for effective postdischarge support strategies [15].

In this review, the term FCC refers to family-centered communication, defined as structured, intentional, and collaborative communication strategies delivered by healthcare professionals to engage families of preterm infants in the care process. These strategies include structured health education, counseling, skills training, multidisciplinary team consultations, and active family involvement in decision-making. This concept is distinct from Family-Centered Care, which refers to an organizational philosophy and institutional policy framework [16]. The present review focuses on communication-based strategies designed to enhance caregiver competence and improve preterm infant health outcomes.

FCC has emerged as a potentially viable approach to improve primary caregivers’ knowledge, attitudes, and proficiency in delivering home care for newborns. This method prioritizes cooperation among healthcare professionals and families, integrating interventions including health education, counseling, and active family participation in clinical decision-making [17]. Existing research suggests this strategy may be efficacious in augmenting family caregiving ability (enhancing caregivers’ knowledge, attitudes, and skills), which in turn may lead to improved health outcomes for preterm infants (including overall health status, growth and developmental milestones, fewer complications, and lower hospitalization rates) and greater family satisfaction with the care process [18–20].

Evidence suggests that structured family-centered communication–based interventions implemented in neonatal care settings are associated with improved maternal discharge preparedness, reduced stress and anxiety, and enhanced caregiver education [21]. This may have an additional effect on the reduction in readmission rates within the first 6 months following discharge from the NICU [22]. Furthermore, parental confidence in managing high-risk infant conditions has been reported to increase substantially as a result of skills training that emphasizes communication through hospital simulations [23]. Other research has also demonstrated that group-based FCC programs, which provide families with support through joint training sessions, can enhance social interaction and alleviate family tension [24]. It has been suggested that enhanced caregiver education, attitudes, and competency may reduce the potential severity of conditions in preterm infants [19], potentially resulting in improved health conditions, growth and development, and reduced hospital length of stay, which may be followed by reduced readmission rates [25].

Nevertheless, while these findings are encouraging, additional comprehensive research is needed to investigate the implementation of these programs, including their design, delivery methods, health information content, duration of intervention, and the comparative effectiveness of various FCC components (including health education and counseling, communication skills training, multidisciplinary team consultations, and active family involvement in infant care) compared to standard care in enhancing caregiving capacity and health outcomes for preterm infants.

This review also highlights the need for a more comprehensive evaluation of outcomes to ensure the successful implementation of FCC. Given the critical role of caregiving ability in shaping preterm infant health outcomes, a detailed assessment of both intervention types and measured endpoints is essential. This evaluation provides a thorough understanding of the efficacy of FCC in enhancing caregiver capacity and improving the clinical and developmental trajectories of premature infants.

A preliminary search of the International Prospective Register of Systematic Reviews (PROSPERO), PubMed, the Cochrane Database of Systematic Reviews, and JBI Evidence Synthesis revealed that no systematic review has specifically evaluated the impact of FCC on both caregiving ability and infant health outcomes in preterm populations, nor are there any ongoing or completed reviews addressing this combined PICO framework.

This review aims to assess the effectiveness of FCC approaches versus standard care in terms of (1) the ability to provide care—caregivers’ knowledge, attitudes, and skills—and 2) infant health outcomes—overall health, growth and developmental progress, incidence of complications, and hospitalization rates among mothers or primary caregivers of preterm infants in various healthcare and community settings. By synthesizing global evidence, the systematic review will inform optimal FCC implementation strategies, guide policy-making, and ultimately help to alleviate the global burden of preterm infant morbidity and mortality.

## Research questions

The specific research questions are as follows: *What is the effectiveness of FCC strategies in improving caregiving abilities—specifically caregivers’ knowledge, attitudes, and skills—and enhancing health outcomes—including overall health status, growth and developmental milestones, reduced complications, and hospitalization rates—for preterm infants and their mothers or primary caregivers?*

## Methods/design

This systematic review protocol aims to assess the effectiveness of family-centered communication on caregiving capacity and health outcomes of preterm infants. This protocol is not an amendment of a previous publication. Future important amendments will be duly documented. The protocol will comply with the Preferred Reporting Items for Systematic Review and Meta-Analysis Protocols and Systematic Reviews and Meta-Analyses (PRISMA) checklist [26, 27] (see Additional file 1).

The protocol was developed and checked against the PRISMA-P (Preferred Reporting Items for Systematic Review and Meta-Analysis Protocols) checklist, which is provided as Additional file 1. The planned study identification, screening, eligibility assessment, and inclusion process will follow the PRISMA framework to ensure methodological transparency.

The PICO framework (Participants, Intervention, Comparator, Outcomes) was used operationally to (1) define eligibility criteria, (2) construct search strings and indexing terms, and (3) design the title/abstract and full-text screening and data-extraction forms. At the reporting stage of the completed review, the PRISMA 2020 checklist and flow diagram will be applied and included in the final report.

### Eligibility criteria

The eligibility criteria for this review were structured via the Participants, Interventions, Comparators, and Outcomes (PICO) framework to ensure a systematic and transparent selection of relevant studies. To ensure methodological rigor and contextual relevance, this stage will be conducted by two independent reviewers, with expert input from a neonatologist (TT) at Hasan Sadikin Hospital and two family medicine specialists (HR and IN).

### Participants

This review will encompass studies focusing on preterm infants and their mothers or primary caregivers, with consideration given to all gestational subcategories, including extremely preterm, very preterm, and moderate to late preterm infants. Studies conducted in any context will be deemed eligible for inclusion. Conversely, studies involving preterm infants with co-occurring comorbidities or complications were excluded from the review. The exclusion of preterm infants with co-occurring comorbidities will aim to reduce clinical heterogeneity and confounding, enabling a more focused assessment of the effects of family-centered communication on caregiving capacity and infant health outcomes among clinically stable preterm populations. The implications of this exclusion for generalizability will be explicitly considered when interpreting the findings, and future research is warranted to examine FCC interventions in more clinically complex preterm groups. Studies set in any context (e.g., hospital neonatal units, primary health centers, home care, community settings) will be eligible.

### Interventions

This review will include studies evaluating Family-Centered Communication strategies implemented by healthcare professionals and include health education and counseling, communication skills training, multidisciplinary team consultations, and active family involvement in infant care. These interventions can be delivered alone or alongside standard care. Healthcare professionals delivering the FCC intervention include those directly involved in maternal and newborn care and family support—such as midwives, nurses, pediatricians, general practitioners, clinical psychologists, and other multidisciplinary healthcare providers—who are trained and authorized to deliver health education, counseling, and structured communication interventions.

To enhance cross-study comparability, FCC interventions will be analytically characterized according to their core components, including communication modality (e.g., face-to-face, digital, or blended), caregiver engagement strategy (e.g., education, counseling, shared decision-making), delivery mechanism (individual or group-based), intensity, and implementation setting. Interventions sharing similar functional components and outcome constructs will be grouped for synthesis, while those with substantially different profiles will be analyzed separately or synthesized narratively.

### Comparators

The review will consider any comparator, including standard care without an FCC component, alternative family-based communication strategies, and typical home-visit or routine health education programs.

### Outcomes

Studies must report on at least one of the following domains: caregiving ability and infant health outcomes. Caregiving ability is defined as the primary outcome, while infant health outcomes are considered secondary outcomes. The evaluation of caregivers’ ability, including knowledge, attitudes, and skills, will be conducted via validated instruments, including but not limited to. The Premature Birth Knowledge Scale (PB-KS) is used to assess knowledge of infant care [28], the Self-Efficacy in Infant Care Scale (SICS) is used to assess maternal judgments about the ability to care for the baby [29], and the Caring Ability of Mother with Preterm Infant Scale (CAMPIS) is used to evaluate caregiving competencies [30] These instruments are included to guide the identification and extraction of outcomes related to caregivers’ cognitive, affective, and behavioral domains. For example, PB-KS captures informational understanding of premature infant needs, SICS measures mothers’ perceived confidence and self-efficacy in caregiving, and CAMPIS assesses the practical and emotional components of caregiving ability. Studies using equivalent or adapted validated tools will also be included to ensure comprehensive outcome synthesis.

Assessment of the health status of infants will be conducted via validated instruments, including but not limited to internationally recognized standards. Growth will be evaluated via the WHO Growth Chart, which enables the monitoring of physical development such as weight, length, and head circumference according to age. Development will be measured via the Bayley Scales of Infant and Toddler Development (BSID) [31], which provides a comprehensive evaluation of the cognitive, motor, and behavioral aspects of infants and toddlers. Medical complications will be assessed with the Neonatal/Infant Health and Complication Scale, allowing for the identification and assessment of various neonatal and infant complications, including respiratory issues, infection, and metabolic disorders. Finally, hospitalization rates will be measured using hospitalization rate scales, which evaluate the frequency and duration of hospital stays and their impact on infant health status. These tools will ensure that the evaluation is carried out on the basis of evidence-based methods and internationally recognized standards.

To address potential disparities in outcome measures and follow-up periods, outcomes will be grouped into standardized domains reflecting comparable underlying constructs, irrespective of the specific instruments used. Where different validated tools measure the same construct, effect sizes will be standardized using the standardized mean difference (SMD). Outcomes that are conceptually incomparable will not be pooled. Variability in follow-up duration will be explicitly considered during synthesis, with analyses conducted within comparable timeframes where feasible, or otherwise synthesized narratively following the SWiM framework. No restrictions on follow-up duration will be applied; time points such as discharge, 3 months, 6 months, and 12 months will be included where reported.

This review will include both experimental and quasi-experimental designs (including randomized controlled trials, nonrandomized controlled trials, and before-and-after studies), as well as analytical observational designs (prospective and retrospective cohort studies, and case–control studies). The inclusion of multiple study designs aims to capture comprehensive evidence on the effectiveness and implementation of FCC interventions, which are behavioral and communication-based in nature and may not always be feasible to evaluate through randomized trials alone due to ethical and logistical constraints. Experimental studies will be prioritized for causal inference, while observational studies will provide contextual understanding of real-world application, sustainability, and generalizability.

Editorials, opinion pieces, conference abstracts without full-text publications, and qualitative studies were excluded as they do not provide extractable quantitative effect estimates required to address the review objectives and support the planned quantitative synthesis and GRADE-based certainty assessment.

### Search strategy

The search strategy will aim to locate published studies only. An initial limited search of PubMed and Scopus was undertaken to identify articles on the topic. The text words contained in the titles and abstracts of relevant articles, along with the index terms used to describe them, were used to develop a comprehensive search strategy for PubMed, (see in Table 1). To ensure a comprehensive search, a manual screening of high-impact journals will be conducted to identify additional studies relevant to the research question [32].

**Table 1:** Search strategy for MEDLINE (PubMed), conducted March 12, 2025

Concept	Query	Records retrieved
#1	“infant, premature”[MeSH Terms] OR “preterm”[Title/Abstract] OR “preterm infant”[Title/Abstract] OR “premature baby”[Title/Abstract] OR “preemie”[Title/Abstract]	139,812
#2	“communication”[MeSH Terms] OR “ounselling”[Title/Abstract] OR “health promotion” [Title/Abstract] OR “parent provider communication”[Title/Abstract] OR “family centered communication”[Title/Abstract]	526,946
#3	“growth and development”[MeSH Terms] OR “health indicators”[Title/Abstract] OR “morbidity”[Title/Abstract] OR “mortality”[Title/Abstract] OR ((“infant, newborn”[MeSH Terms] OR (“infant”[All Fields] AND “newborn”[All Fields]) OR “newborn infant”[All Fields] OR “neonatal”[All Fields] OR “neonate”[All Fields] OR “neonates”[All Fields] OR “neonatality”[All Fields] OR “neonatals”[All Fields] OR “neonate s”[All Fields]) AND “indicators health”[Title/Abstract])	2,941,768
#4	("health knowledge, attitudes, practice"[MeSH Terms] OR "caregivers"[Title/Abstract] OR "caregiving"[Title/Abstract]) AND (english[Filter])	224,169
#5	#1 AND #2 AND #3 AND #4	843
Limited to English		

Studies published in English will be included. No publication date restrictions will be applied. This decision aims to ensure the inclusion of all relevant evidence concerning FCC in the care of preterm infants, recognizing that this concept has evolved over time. Including both early and recent studies allows a more comprehensive understanding of how FCC approaches have developed and been implemented in different eras. To mitigate potential temporal bias, all included studies will be critically appraised for contextual relevance, methodological quality, and applicability to contemporary healthcare practice. Older studies employing outdated FCC frameworks will be discussed narratively with interpretive caution. Electronic database searches will be conducted in PubMed (NCBI), EMBASE (Elsevier), Scopus (Elsevier), and CINAHL (EBSCOhost). ScienceDirect (Elsevier) will be used as a supplementary source for full-text retrieval when necessary but is no longer the primary database. EMBASE and CINAHL were added to improve capture of biomedical and nursing/midwifery practice literature that may not be fully indexed in PubMed or Scopus, while PsycINFO was included to better capture psychological and communication-related aspects of caregiving. In addition to electronic searches, reference lists of included studies and relevant reviews will be hand-searched, and forward citation searches will be performed.

We will include peer-reviewed studies published in English. Grey literature (e.g., technical reports, theses/dissertations, conference abstracts not published as full-text papers) and studies published in languages other than English will not be included due to resource constraints for translation and quality appraisal. To mitigate the potential bias introduced by these exclusions, we will: (1) perform reference-list checks and forward citation searches; (2) record and report the number of non-English records identified and excluded in the PRISMA flow diagram; and (3) assess potential publication bias quantitatively (where feasible) and discuss these limitations in the Discussion.

The search strategy was developed collaboratively by the review team, guided by the PRESS 2015 (Peer Review of Electronic Search Strategies) guideline recommendations [33]. Although the strategy has not yet undergone a formal peer review by an information specialist, it was internally validated by the review team for logical completeness, use of appropriate Boolean operators, and alignment with database-specific indexing. This will be acknowledged as a methodological limitation, and librarian input may be sought in the final implementation stage to further enhance search precision and comprehensiveness.

Search strategy, study selection, critical appraisal, and data extraction will be conducted independently by two reviewers (MM and QESA). Any disagreements arising at any stage will be resolved through discussion, and where consensus cannot be reached, senior reviewers (DA and DH) will be consulted to arbitrate and make the final decision [32].

Across all stages of the review process, two reviewers (MM and QESA) will act as primary independent assessors for study identification, screening, data extraction, and quality appraisal.

Where required data are missing, unclear, or insufficiently reported, attempts will be made to contact the corresponding authors of the original studies via email, with up to two contact attempts. If no response is received, available data will be used, and the potential impact of missing information will be addressed in the synthesis and discussion.

For studies employing cluster randomized designs, appropriate cluster-level or baseline covariate adjustments reported by the original study will be considered where available. The covariates may include gestational age, birth weight, neonatal comorbidities or baseline severity, maternal characteristics such as age and education, and healthcare context, as these are clinically relevant confounders that may influence both exposure to FCC and infant or caregiver outcomes. No additional data-driven covariate selection will be undertaken by the review team; where such adjustments are not reported or statistical correction is not feasible, the study will be synthesized narratively. Crossover trials will be included where relevant, with consideration given to potential carryover effects, and only appropriate data (e.g., first-period data) will be used where necessary.

Any disagreements at any stage will be resolved through discussion, and where consensus cannot be reached, senior reviewers (DA and DH) will be consulted to arbitrate and make the final decision. This predefined allocation of roles is intended to ensure consistency, transparency, and methodological rigor throughout the review process.

### Study selection

Following the search, all identified citations will be collated and uploaded into Rayyan**.** Duplicates will be automatically checked using Rayyan’s built-in deduplication feature, and identified duplicates will be re-examined and removed to maintain focus in the screening process. All articles obtained from the selected databases will be exported in RIS or BibTeX format and imported into Rayyan. The platform’s compatibility with various file formats ensures seamless article integration, minimizing the risk of losing relevant studies.

After the pilot test, two independent reviewers (MM and QESA) will screen the titles and abstracts to assess eligibility based on the predefined inclusion criteria. The pilot test ensures that the inclusion and exclusion criteria are clearly defined and consistently applied by both reviewers. Any disagreements will be resolved through discussion, and where consensus cannot be reached, senior reviewers (DA and DH) will be consulted.

If the review authors’ own work may be eligible for inclusion, appropriate safeguards, such as abstaining from study selection, will be implemented to mitigate potential conflicts of interest. Studies deemed potentially relevant will be flagged for full-text screening, and citation details will be imported into Rayyan for further evaluation.

Full-text articles will be independently assessed against the inclusion criteria by two reviewers (MM and QESA). Reasons for exclusion of full-text studies that do not meet the inclusion criteria will be documented and reported in the systematic review. Any disagreements arising during the study selection process will be resolved through discussion, or, where consensus cannot be reached, by consultation with senior reviewers (DA and DH). The results of the search and study selection process will be fully reported in the final systematic review, including a PRISMA flow diagram (26).

The study selection process will be documented and presented using the PRISMA flow diagram to ensure transparency and reproducibility. The diagram will include the number of records identified from each database and additional sources (e.g., handsearching and citation tracking), duplicates removed, titles and abstracts screened, full-texts assessed for eligibility, and studies included in the review. Reasons for the exclusion of full-text studies will be systematically recorded and summarized. Non-English and grey literature records identified but excluded will also be noted, and all screening decisions will be tracked in Rayyan, which provides an exportable screening log to support transparent reporting.

### Data extraction

Data will be extracted from the included studies by two independent reviewers using a standardized data extraction sheet specifically developed for this review [34], implemented in Microsoft Excel. The extraction sheet will be pilot-tested on a subset of 3–5 eligible studies of various designs to ensure clarity, consistency, and comprehensiveness of extraction items. During the pilot phase, two reviewers (MM and QESA) will independently extract data and compare their results to identify discrepancies or ambiguities in the interpretation and application of extraction fields. Any discrepancies will be resolved through discussion, or, if necessary, by consultation with senior reviewers (DA and DH). Feedback from this pilot testing will inform modifications and iterations of the extraction sheet, including rewording unclear items, adding relevant variables, and removing redundancies. The finalized version of the data extraction form will be agreed upon by consensus within the review team and provided as supplementary material to ensure transparency and reproducibility.

For the full data extraction process, two reviewers will independently extract data using the finalized tool, and any discrepancies will be resolved through discussion or, if necessary, consultation with a third reviewer [34]. The extracted data will include: (1) study characteristics (authors, year, country, and study design), (2) population details (infant gestational age, maternal demographics, and caregiver roles), (3) intervention specifics (type of FCC, delivery mode, frequency, and duration), (4) outcomes (caregiving capacity, including knowledge, attitudes, and skills, as well as infant health outcomes such as growth and morbidity), and (5) statistical information (effect sizes, confidence intervals, and p-values) [32, 34]. Study characteristics will be summarized descriptively, with continuous variables reported as means and standard deviations or medians and interquartile ranges, as appropriate, and categorical variables presented as frequencies and percentages. The study authors will be contacted up to two times to request missing or additional data when required.

### Quality assessment

Risk-of-bias appraisal tools were selected to ensure methodological appropriateness across study designs, with ROBINS-I applied to non-randomized intervention studies and the Newcastle–Ottawa Scale used for analytical observational designs.

The methodological quality and risk of bias of the included studies will be evaluated using appropriate standardized tools on the basis of study design. Risk of bias will be independently assessed by two reviewers using design-specific appraisal tools: (1) For randomized controlled trials (RCTs), the Cochrane Risk of Bias 2.0 (RoB 2) tool will be used, evaluating bias across five domains: randomization, deviations from intended interventions, missing outcome data, measurement of outcomes, and selective reporting [35]. The RoB 2 tool will be applied in its standard form, with particular attention to domains relevant to communication-based interventions, including deviations from intended interventions and outcome measurement. (2) For quasi-experimental or nonrandomized intervention studies, the ROBINS-I (Risk Of Bias In Nonrandomized Studies—of Interventions) tool will be employed to assess confounding, comparability between groups, and bias in outcome measurement [36]. In line with ROBINS-I guidance, risk of bias will be assessed at the level of each relevant outcome within individual studies. These outcome-level assessments will be interpreted alongside study-level quality appraisals (e.g., Newcastle–Ottawa Scale) to provide a comprehensive and triangulated evaluation of methodological quality and risk of bias. (3) For observational analytical studies, including cohort and cross-sectional designs, the Newcastle–Ottawa Scale (NOS) will be applied to appraise study quality based on sample selection, comparability, and outcome assessment [37]. Results of the risk of bias assessments will be summarized in tabular form and will inform interpretation of findings and sensitivity analyses. Two reviewers (MM and QE) will independently perform the quality assessment. Any discrepancies will be resolved through discussion, or, where consensus cannot be reached, by consultation with senior reviewers (DA and DH) [38].

### Data analysis

#### Assessment and management of heterogeneity

To address potential heterogeneity, this review adopts a structured, multi-level approach encompassing design-level, analytical-level, and synthesis-level considerations. Statistical heterogeneity will be quantified using the *χ*^2^ test and the *I*^2^ statistic, and explored through predefined subgroup analyses, sensitivity analyses, and, where feasible, meta-regression. At the design level, meta-analyses will be restricted to methodologically comparable studies, and pooling across fundamentally different study designs will not be undertaken. At the analytical level, statistical heterogeneity will be quantified using the *I*^2^ statistic and interpreted in accordance with Cochrane Handbook guidance. Predefined subgroup and sensitivity analyses will be conducted to explore potential sources of heterogeneity informed by theoretical relevance to FCC mechanisms. Where heterogeneity remains substantial or cannot be meaningfully addressed through quantitative synthesis, findings will be synthesized narratively in accordance with the SWiM framework to maintain interpretability and methodological transparency [39].

#### Functional comparability of interventions

For data synthesis, FCC interventions will be compared based on functional equivalence rather than surface characteristics. Interventions will be considered analytically comparable when they share similar underlying communication mechanisms and target similar caregiving or infant health outcomes, even if delivery formats differ.

#### Effect measures and data transformation

Effect estimates will be derived according to outcome type and measurement scale. Mean differences (MD) will be used for continuous outcomes measured on the same scale, and standardized mean differences (SMD) when different validated instruments assess the same construct. For dichotomous outcomes, risk ratios (RR) or odds ratios (OR) with 95% confidence intervals will be applied. Where necessary, estimates will be transformed to a common metric in line with Cochrane Handbook guidance [40].

For ratio measures, logarithmic transformation will be applied where appropriate, while continuous outcomes measured using different scales will be standardized using established methods, in accordance with the Cochrane Handbook for Systematic Reviews of Interventions [40].

#### Handling of adjusted estimates and confounding

Adjusted estimates will be prioritized when available, and quantitative pooling will be restricted to studies with conceptually comparable adjustment sets. Adjusted estimates are prioritized as they better account for confounding factors that are highly relevant to preterm infant outcomes, including gestational age, birth weight, neonatal comorbidities, maternal characteristics, and healthcare context. By controlling for these factors, adjusted estimates provide more robust and clinically interpretable effect estimates, particularly in observational and quasi-experimental studies where randomization is not feasible. Where adjustment strategies differ substantially or are insufficiently reported, findings will be synthesized narratively following the SWiM framework.

#### Criteria for quantitative synthesis

Where possible, studies will be pooled using statistical meta-analysis in Review Manager (RevMan, Copenhagen: The Nordic Cochrane Centre). Meta-analysis will be undertaken only when at least two studies are sufficiently comparable in terms of population, outcome constructs, and follow-up period. Where methodological, conceptual, or temporal comparability is not met, findings will be synthesized narratively in accordance with the SWiM framework. Effect sizes will be reported as mean differences or standardized mean differences for continuous data, and risk ratios or odds ratios for categorical data, with 95% confidence intervals [32, 34]. To ensure consistency, different effect measures (RR, OR, RD, MD, SMD) will be converted to a common metric where appropriate. For ratio measures, log transformation will be applied prior to pooling, and continuous outcomes measured on different scales will be standardized using the standardized mean difference, in accordance with the Cochrane Handbook for Systematic Reviews of Interventions. Outcomes measuring similar constructs will be grouped into standardized categories. Outcomes that are conceptually incomparable will be excluded from meta-analysis and presented narratively.

Grouping outcomes into standardized categories will be based on conceptual mapping to shared underlying constructs rather than statistical harmonization across fundamentally different measures. Non-collapsible effect measures (e.g., odds ratios or hazard ratios) will not be converted across effect types and will be synthesized within their respective categories or narratively where appropriate.

Pooling will only be undertaken when adjustment sets are conceptually comparable. Conversions between fundamentally different effect measures (e.g., hazard ratios and risk ratios) will not be performed. Where effect measures are non-collapsible or adjustment strategies vary substantially, quantitative synthesis will be avoided and findings will be synthesized narratively following the SWiM framework.

Variability in follow-up duration is anticipated and will be addressed during synthesis. Where feasible, outcomes will be analyzed within comparable follow-up intervals commonly reported in neonatal research (≤28 days, ≤6 months, and ≤12 months). Quantitative pooling will be restricted to studies with similar follow-up durations; otherwise, findings will be synthesized narratively following the SWiM framework.

#### Exploration of sources of heterogeneity

Statistical heterogeneity will be assessed using the *χ*^2^ test and the *I*^2^ statistic. A fixed-effects model will be applied when *I*^2^ ≤ 50%, and a random-effects model will be used when *I*^2^ > 50% [32, 34].

Model selection will be guided by both statistical indicators and conceptual heterogeneity, with random-effects models preferred when heterogeneity is expected based on differences in study design, populations, interventions, or outcome measurement.

In addition to statistical assessment of heterogeneity using the *χ*^2^ and *I*^2^ statistics, potential sources of heterogeneity will be explored conceptually and analytically.

Subgroup analyses and, where feasible, meta-regression will be conducted to identify contributors such as differences in case mix (e.g., infant gestational age or comorbidities), maternal and caregiver characteristics (e.g., age, education, and caregiving roles), intervention features (type of FCC, delivery mode, duration, and frequency), outcome measurement, and contextual factors (e.g., healthcare setting, resource availability, and regional differences between high- and low-income countries). Meta-regression will be undertaken only when a sufficient number of studies are available per covariate, and the number of moderators included will be restricted to a small set selected based on theoretical relevance to FCC mechanisms and empirical feasibility.

When substantial heterogeneity (*I*^2^ > 50%) is detected, a random-effects model will be used, and subgroup or sensitivity analyses will be undertaken to assess the robustness of findings. Where pooling is not appropriate, findings will be presented narratively following the SWiM framework.

Given the inclusion of diverse study designs, meta-analyses will be performed only within homogeneous groups of studies with comparable methodology and outcome measures (e.g., randomized controlled trials analyzed separately from observational or quasi-experimental studies) to maintain internal validity. Pooling of data across fundamentally different study designs will not be undertaken. Potential design-related biases including recall bias in case–control studies, temporal bias in cross-sectional studies, and confounding in cohort or quasi-experimental designs—will be critically considered during data synthesis.

Subgroup analyses will be conducted, where data permit, to explore variability by population characteristics (e.g., infant age, caregiver type), intervention type, and intensity. Sensitivity analyses will assess the robustness of pooled results by excluding studies with high risk of bias or statistical outliers.

#### Narrative synthesis (SWiM Framework)

When statistical pooling is not appropriate due to substantial heterogeneity, limited data, or fundamental design differences, findings will be synthesized narratively in accordance with the Synthesis Without Meta-analysis (SWiM) guideline [39]. Reporting will follow the SWiM framework to ensure transparency and methodological rigor.

#### Publication bias assessment

Publication bias will be assessed using funnel plots in RevMan when at least ten studies are available, and Egger’s test will be used to evaluate funnel plot asymmetry where appropriate.

#### Certainty of evidence (GRADE)

The Grading of Recommendations, Assessment, Development and Evaluation (GRADE) approach will be applied to assess the certainty of evidence. A Summary of Findings table will be developed using GRADEpro GDT (McMaster University, ON, Canada) [34], presenting absolute and relative effects, confidence intervals, and an overall rating of evidence certainty based on risk of bias, directness, heterogeneity, precision, and publication bias. The outcomes evaluated will include parental knowledge, attitudes, and caregiving skills; and infant outcomes such as health status, growth, developmental milestones, complication rates, and hospitalizations.

Certainty of evidence will be assessed per outcome, starting at “high” for randomized studies and “low” for observational studies, and may be downgraded to “moderate”, “low”, or “very low” based on GRADE domains including risk of bias, inconsistency, indirectness, imprecision, and publication bias, or upgraded where appropriate (e.g., large effect or dose–response). Results from the RoB 2, ROBINS-I, and NOS assessments will directly inform the risk-of-bias domain in GRADE.

**Fig. 1:**
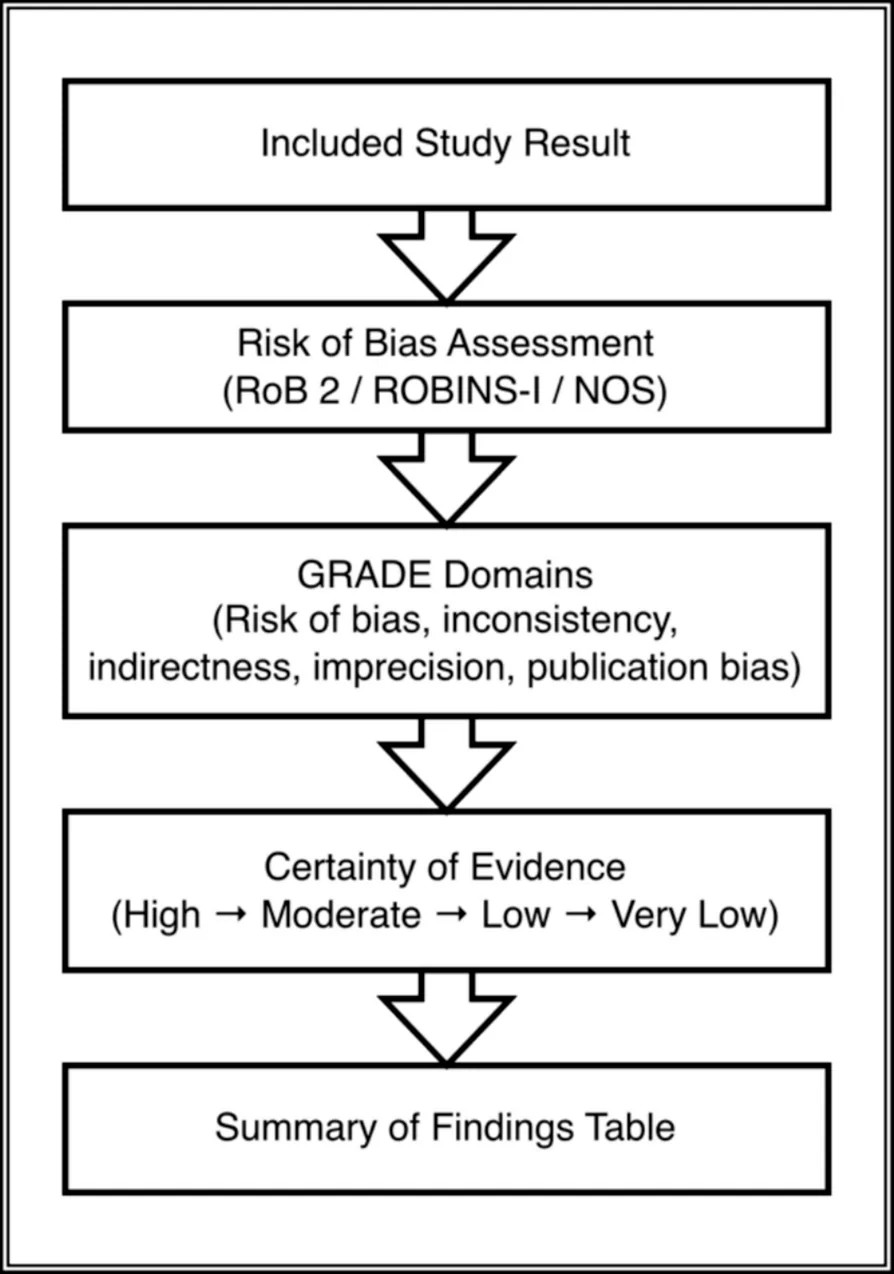
Application of the GRADE framework for assessing certainty of evidence in this systematic review

Figure 1 illustrates the GRADE framework and summarizes how risk-of-bias assessments, consistency, directness, precision, and publication bias inform certainty ratings and the development of the Summary of Findings table.

It is acknowledged that when multiple study designs are synthesized, residual heterogeneity may persist due to differences in methodological quality, measurement approaches, and contextual factors, which may not be fully captured even under a random-effects model.

## Discussion

This systematic review is designed to critically assess the effectiveness of FCC strategies in comparison with standard care in improving the health outcomes of preterm infants and strengthening the caregiving capacities of families within various healthcare and community contexts. By consolidating the most reliable evidence, the review seeks to offer actionable recommendations for healthcare providers, policymakers, and program designers in the development and implementation of FCC-based interventions. The anticipated outcomes are expected to inform evidence-based approaches that support the early developmental trajectories of preterm infants and enhance family participation in care, thereby contributing to improved health and developmental outcomes in both the short and long term. By synthesizing global evidence, this review seeks to inform optimal FCC implementation strategies, guide policy-making, and ultimately help to alleviate the global burden of preterm infant morbidity and mortality. By integrating evidence from diverse global contexts, this review aims to generate insights that support the effective implementation of FCC strategies, inform evidence-based policy development, and contribute to reducing the global burden of morbidity and mortality associated with preterm birth.

Nevertheless, several methodological challenges may be encountered throughout the review process. One major concern is the expected heterogeneity in the design of the included studies—spanning randomized controlled trials, quasi-experimental designs, and observational research—which may complicate the data synthesis process, especially with respect to conducting meta-analyses. Variations in FCC intervention components, such as differences in communication modalities, strategies for caregiver engagement, and delivery mechanisms, may further reduce cross-study comparability. Additionally, disparities in outcome measures and follow-up periods—particularly those related to caregiver competence and infant health indicators—could limit the capacity to formulate consistent and generalizable conclusions. There is also a risk of language and publication bias, particularly if eligible studies are published in non-English languages or in sources not indexed in mainstream academic databases. To mitigate these challenges, this review will employ a robust and transparent methodological framework, including independent review by multiple assessors, the use of validated critical appraisal instruments, and, where appropriate, the application of subgroup analyses to explore potential sources of heterogeneity.

## Supplementary Information

Additional file 1: PRISMA checklist.

## Data Availability

All data relevant to this systematic review protocol, including search strategies, inclusion/exclusion criteria and data extraction forms are available from the corresponding author upon reasonable request. Any data used in the review will be made available in accordance with the transparency and reproducibility standards of the journal.

## Abbreviations

BSID: Bayley Scales of Infant and Toddler Development
CAMPIS: Caring Ability of Mother with Preterm Infant Scale
CI: Confidence interval
CINAHL: Cumulative Index to Nursing and Allied Health Literature
EMBASE: Excerpta Medica Database
FCC: Family-Centered Communication
GRADE: Grading of Recommendations, Assessment, Development and Evaluation
GRADEpro GDT: GRADEpro Guideline Development Tool
JBI: Joanna Briggs Institute (JBI Evidence Synthesis)
MD: Mean difference
NICU: Neonatal intensive care unit
NOS: Newcastle–Ottawa Scale
OR: Odds ratio
PB-KS: Premature Birth Knowledge Scale
PICO: Participants, Intervention, Comparator, Outcomes
PRISMA: Preferred Reporting Items for Systematic Reviews and Meta-Analyses
PRISMA-P: Preferred Reporting Items for Systematic Review and Meta-Analysis Protocols
PROSPERO: International Prospective Register of Systematic Reviews
RD: Risk difference
RevMan: Review Manager (software by The Nordic Cochrane Centre)
RoB 2: Risk of Bias 2 (revised Cochrane tool for randomized trials)
ROBINS-I: Risk Of Bias In Nonrandomized Studies — of Interventions
RCT: Randomized controlled trial
RR: Risk ratio
SICS: Self-Efficacy in Infant Care Scale
SMD: Standardized mean difference
SWiM: Synthesis Without Meta-analysis (guidelines for structured narrative synthesis)
UNICEF: United Nations Children’s Fund
WHO: World Health Organization
